# Non-contrast *trans*-catheter aortic valve implantation using Evolut self-expanding platform: a wire backup technique case report

**DOI:** 10.1093/ehjcr/ytag366

**Published:** 2026-05-19

**Authors:** Krishnarpan Chatterjee, Mohamed Ali, Mohammad Alkhalil

**Affiliations:** Cardiothoracic Centre, Freeman Hospital, Freeman Road, Newcastle-upon-Tyne NE7 7DN, UK; Cardiothoracic Centre, Freeman Hospital, Freeman Road, Newcastle-upon-Tyne NE7 7DN, UK; Cardiothoracic Centre, Freeman Hospital, Freeman Road, Newcastle-upon-Tyne NE7 7DN, UK; Translational and Clinical Research Institute, Newcastle University, Newcastle-upon-Tyne NE1 7RU, UK

**Keywords:** Aortic stenosis, Transcatheter aortic valve implantation, Renal impairment, Self-expanding, Evolut, Case report

## Abstract

**Background:**

Concomitant aortic stenosis and chronic kidney disease (CKD) represent management challenges. Transcatheter aortic valve implantation (TAVI) is an established treatment for patients with symptomatic severe aortic stenosis but requires the use of contrast media, particularly in patients undergoing self-expanding platform implantation. Therefore, patients with CKD remain at an increased risk of developing worsening renal impairment post TAVI.

**Case summary:**

Herein, we present a 62-year-old male patient who was admitted with progressive dyspnoea. His transthoracic echocardiogram revealed severe aortic stenosis and impaired left ventricular systolic function (an estimated ejection fraction of 35%). He has a background of porcelain aorta and CKD. Despite low-dose contrast CT, the patient’s renal function significantly deteriorated. He was successfully treated with *trans*-catheter aortic valve implantation using a self-expanding valve (Evolut) without the need for contrast media. The procedure was guided by catheter positioning in the non-coronary cusp and the wire back-up technique in the left coronary cusp.

**Conclusion:**

This case demonstrated the feasibility of implanting a self-expanding *trans*-catheter heart valve using the Evolut platform without the need for the use of contrast media in a patient at high risk of worsening renal impairment. The current approach requires good procedural planning and additional vascular access and should be used only in highly selected cases.

Learning points

- Zero-contrast *trans*-catheter aortic valve implantation (TAVI) using a self-expanding platform is feasible in highly selected cases.
- This technique requires additional vascular access and a good understanding of anatomical landmarks during valve deployment.

## Introduction

Patients with chronic kidney disease (CKD) have a higher prevalence of aortic valve disease, with almost a 3-fold increase in the frequency of aortic stenosis compared to those without CKD.^1^ The progression of aortic stenosis in patients with CKD is rapid and unpredictable and is associated with worse short and long-term outcomes after aortic valve replacement.^2^

Trans-catheter aortic valve implantation (TAVI) is an established treatment for patients with severe aortic stenosis.^3^ However, the use of contrast media during TAVI may result in worsening renal impairment in patients with CKD. Previous reports highlighted the feasibility of performing TAVI without contrast using balloon-expandable as well as self-expanding platforms.^4,5^ However, optimal positioning of the *trans*-catheter heart valve remains uncertain without contrast.

## Case presentation

A 62-year-old male patient presented with progressive breathlessness and decompensated heart failure. He has a background of CKD stage 3 (baseline creatinine level of 157 µmol/L) and a previous coronary artery bypass graft. His work-up revealed severe tri-leaflet aortic stenosis with a mean gradient of 44 mmHg and severe left ventricular dysfunction with an estimated ejection fraction of 35%. He underwent computed tomography (CT), which confirmed porcelain aorta, prohibiting surgical intervention following Heart Team discussion, particularly with predicted mortality of 11.2% using the Euro Score II. The patient was subsequently scheduled for *trans*-femoral *trans*-catheter aortic valve implantation (TAVI). Despite low-dose contrast CT, the patient’s renal function significantly deteriorated with a peak creatinine of 525 µmol/L (CKD stage 5). The deterioration in renal function was coupled with a reduction in urine output, but the case did not require renal replacement treatment following discussion with the nephrology team. This was considered to be likely contrast-associated and raised concerns about using contrast during the TAVI procedure.

**Figure 1:**
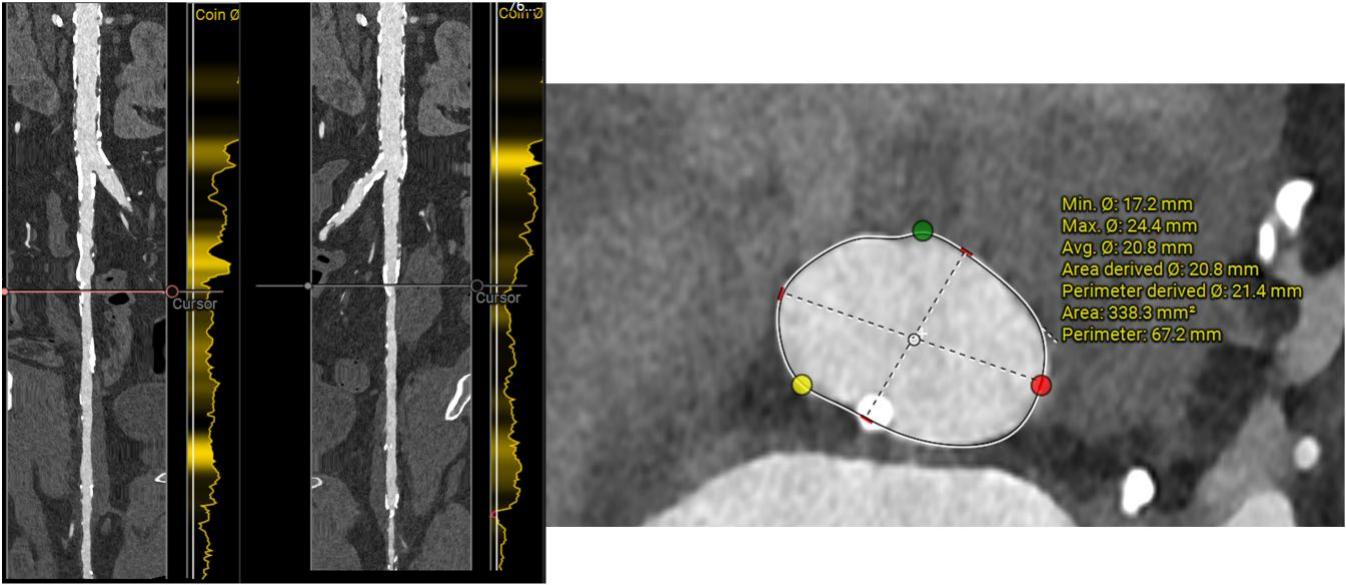
Computed tomography highlighting both the femoral anatomy, as well as the aortic annulus.

The choice of the *trans*-catheter heart valve (THV) was discussed, taking into consideration the small aortic annulus (area of 338 mm^2^ and perimeter of 67 mm), the presence of a calcified nodule at the annular level, and relatively small and calcified femoral arteries (*Figure 1*). These factors favoured the use of self-expanding over balloon-expandable platforms, but at the risk of using contrast media to optimally position the THV. Therefore, a non-contrast TAVI procedure was planned using 26 mm Evolut FX+ (Medtronic, Minneapolis, MN, USA). This was performed using ultrasound-guided puncture of the right common femoral artery. Subsequently, two ProGlides (Abbott Vascular, Santa Clara, CA, USA) were deployed, and an 8-French sheath was inserted to ensure haemostasis. Given the calcified nature of the femoral arteries, intra-vascular lithotripsy using 8.0 mm balloon was used at the outset to deliver the THV.

**Figure 2:**
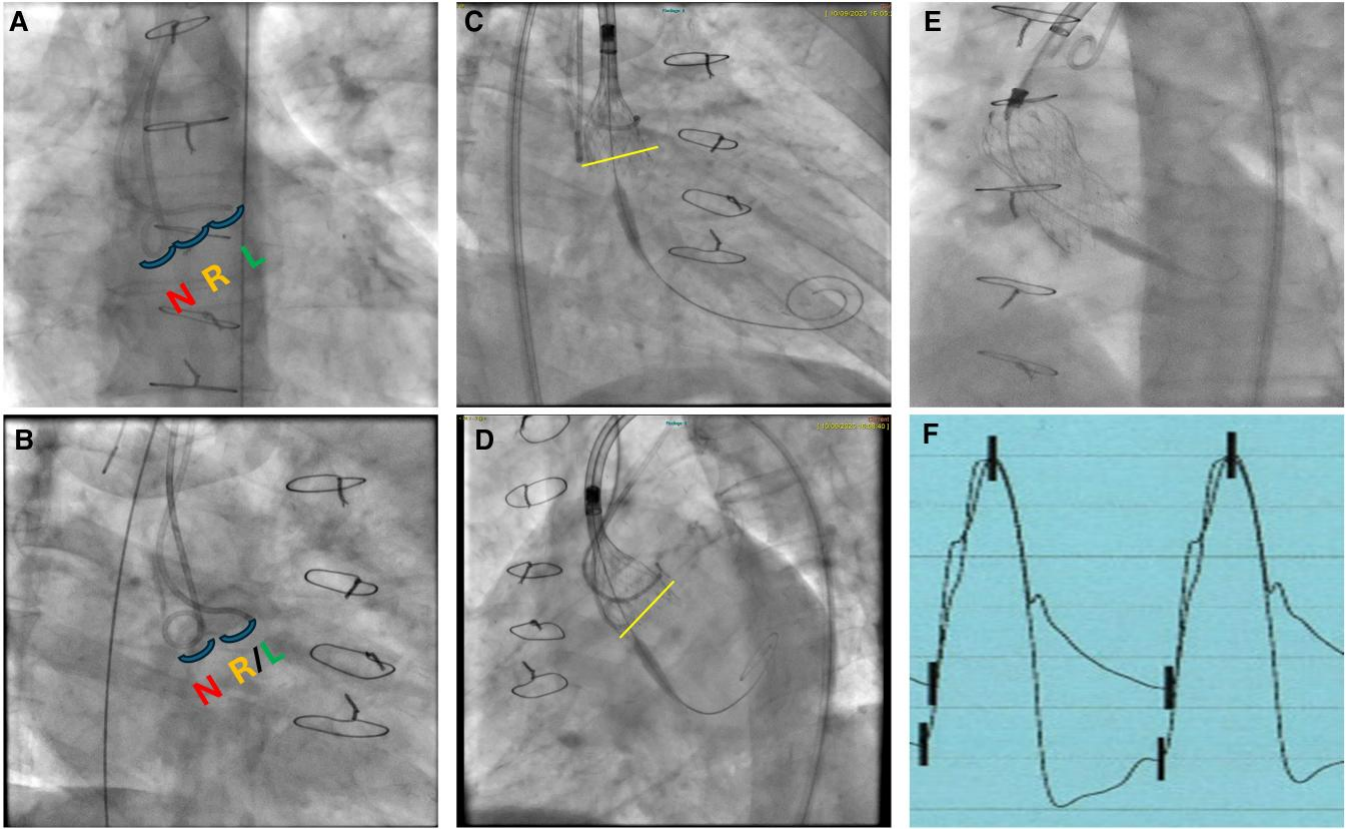
Step-by-step implantation of the Evolut self-expanding platform without contrast use (*Summary Figure*). Diagnostic catheters were positioned in the non- and left coronary sinuses (blue arcs) and confirmed in the cusp overlap (Panel *A*) and co-planar (Panel *B*) views (N, R, and L denote non-, right, and left coronary cusps). The radiopaque gold markers were positioned at the bottom level of the pigtail catheter (the yellow line denotes the annulus level) (Panel *C*). The position of the THV was confirmed in the left anterior oblique (LAO) view by advancing a standard 0.35 wire against the left coronary sinus (Panel *D*). The THV was well expanded (Panel *E*) with excellent invasive haemodynamic results (Panel *F*).

Bi-radial access was obtained. A pigtail catheter was inserted via the right radial artery and positioned in the non-coronary cusp. An amplatz 1 (AL1) catheter was inserted from the left radial artery and positioned in the left coronary sinus (*Figure 2*, Panel *A* & *B*). The position of the catheters was fluoroscopically assessed as highlighted in Panel A.

The THV was advanced over a stiff wire, and using the cusp overlap view (RAO caudal view), the radiopaque gold markers were positioned at the level of the pigtail catheter. The THV valve was subsequently unsheathed and deployed under pacing (*Figure 2*, Panel *C*). During deployment, the AL1 catheter was pushed back, and its relationship with the annular plane was uncertain. This was overcome by pushing a standard 0.035 wire through the AL1 catheter against the left coronary sinus, and the position of the THV was confirmed to be lower than the annulus (*Figure 2*, Panel *D*). The THV was finally released with good expansion (*Figure 2*, Panel *E*). Following deployment, invasive haemodynamic assessment did not reveal any gradient across the valve (*Figure 2*, Panel *F*), and left ventricle end-diastolic pressure was measured as 17 mmHg. Echocardiography revealed trivial aortic regurgitation, with a mean gradient of 7 mmHg (*Figure 2*, Panel *F*). No conduction system disturbances were noted with a stable PR interval of 180 msec and QRS duration of 89 msec. Vascular access was closed in a standard fashion without complications. The patient was monitored using telemetry for 24 h, with no conduction abnormalities during this period. He also underwent daily ECG with no significant change in PR or QRS intervals. The patient was discharged home two days after the procedure with a creatinine level of 105 µmol/L. His heart failure symptoms significantly improved in the 3-month follow-up, with stable renal function and no further admission to the hospital. A summary of the procedural steps is presented in *Table 1*.

**Table 1:** Procedural steps for zero-contrast TAVI using the Evolut self-expanding platform

Procedural setup	Three arterial vascular accesses, including two 6-French and one for the *trans*-catheter heart valve.
Catheters positioning	In the three-cusp view, the pigtail catheter is positioned in the non-coronary cusp, while the AL1 catheter is positioned in the left coronary cusp.
Valve deployment	In the cusp overlap view, the *trans*-catheter heart valve is deployed using the pigtail as a marker of implantation depth. Once the valve is almost at the point of no recapture, the implantation depth is checked in the LAO view.
Assessment of implant depth	Using the gold markers, which are placed at 3 mm above the bottom frame of the Evolut valve, the implant depth can be assessed in the cusp overlap view using the pigtail and in the LAO view using the AL1 catheter. In cases where the AL catheter was misplaced and pushed back by the expansion of the *trans*-catheter heart valve, a 0.035 wire can be advanced into the left coronary sinus to re-establish annular orientation and guide safe valve deployment.

## Discussion

This case demonstrates that optimal planning allowed successful TAVI implantation in a highly challenging patient. Good pre-procedural planning enabled precise positioning of the self-expanding valve without the need to use contrast media in a patient with a high likelihood of developing worsening renal impairment.

Concomitant aortic stenosis and CKD represent diagnostic and management challenges.^2^ Both surgical aortic valve replacement and TAVI are established treatments to manage patients with severe aortic stenosis and CKD. There are limited data comparing clinical outcomes between these two treatment strategies.^6^ TAVI was reported to have lower mortality rates at 30 days, but comparable rates of renal failure and 1-year death with surgery.^6^ Importantly, the patient was considered to have prohibitive surgical risk during the Heart Team discussion. While his predicted mortality was more than 11%, the presence of porcelain aorta added more technical challenges to surgical intervention. Collectively, the consensus was that the percutaneous option with TAVI would offer a safer alternative in a relatively chronologically young but biologically old patient.

The relationship between TAVI and worsening renal function in patients with CKD is complex, and it is not purely related to the use of contrast media. Several factors have also been linked to worsening CKD following TAVI, namely hypotension during valve deployment or secondary to bleeding, and less commonly related to athero-embolic phenomenon.^7^ In the current case, the patient developed worsening renal function following the use of contrast media during CT planning for the TAVI procedure. This phenomenon is relatively uncommon and has recently been reported in 7% of patients undergoing pre-procedural CT planning.^8^ Importantly, contrast use should not be considered as a causal factor but rather associated with worsening renal function in this setting. Other factors, such as contrast volume, baseline renal function, the definition of acute kidney injury, and its timing, would contribute to the variations in the incidence and question any causality link of contrast-associated renal impairment.^8,9^

We elected to avoid the use of contrast during the TAVI procedure. Previous reports highlighted the feasibility of performing TAVI using balloon-expandable, as well as self-expanding platforms.^4,5^ In a case series of 12 patients, Maffeo *et al*. reported favourable outcomes of the TAVI procedure without using contrast media.^4^ However, assessing the depth of implantation, particularly on the left sinus, remains challenging. There were no reliable anatomical landmarks present during fluoroscopy that would allow safe deployment of the THV. Therefore, we used two catheters and positioned them in the non- and left coronary sinus to assist with valve deployment. One challenge we encountered is that the AL1 diagnostic catheter, which was placed in the left coronary sinus, did not remain in position during valve deployment. The use of an end-hole catheter to facilitate advancement of 0.035 wire against the left coronary sinus may enable optimal valve positioning and reflect the true novelty in our case. In other words, the wire served as a marker for the annular plane, and the THV was confirmed to be in good position before valve release. Advancing the wire during valve deployment might be associated with rare risks, particularly given its suboptimal control, including sinus injury, coronary ostial trauma, catheter instability, valve embolization, or arrhythmia. These risks are rare, given that the wire was used in its intended purposes and was advanced under fluoroscopy guidance. Importantly, the wire could have been inserted into the AL1 catheter before unsheathing the THV to function as a stabilizer for the AL1 catheter to prevent dislodgment and maintain its relationship with the annular plane. The choice of THV in our case was carefully considered. The nature of the aortic annulus and femoral arteries favoured the use of SEV with Evolut. The haemodynamic advantage in patients with a small aortic annulus was highlighted in recent studies, including the Small Annuli Randomized to Evolut or Sapien Trial (SMART).^10–12^ Additionally, the presence of a relatively large calcified nodule at the annulus is associated with increased risk of annular rupture using BEV.^13^ Finally, the small size and calcified femoral arteries would support the use of SEV over BEV, given its smaller profile. Notably, the presence of peripheral arterial disease may challenge the use of the *trans*-femoral approach. Other routes, including apical, could have been considered; however, outcomes are likely to be superior using the *trans*-femoral approach.

In conclusion, our case demonstrated the feasibility of implanting a self-expanding *trans*-catheter heart valve using the Evolut platform without the need for the use of contrast media in a patient at high risk of worsening renal impairment. The current approach can only be considered hypothesis-generating and should be used only in highly selected cases.

## Data Availability

Data presented in this case are available from the corresponding author on a reasonable request.

## Lead author biography

Dr Mohammed Alkhalil is an interventional cardiologist and structural heart specialist at the Freeman Hospital, Newcastle-upon-Tyne, UK. He underwent clinical training at the Royal Victoria Hospital in Belfast, obtained his PhD (DPhil) at the University of Oxford, and secured his clinical fellowship at the Toronto General Hospital in Canada.

## Notes

**Consent:** In accordance with COPE guidelines patient informed consent was obtained.

## References

[1] Samad Z, Sivak JA, Phelan M, Schulte PJ, Patel U, Velazquez EJ. Prevalence and outcomes of left-sided valvular heart disease associated with chronic kidney disease. J Am Heart Assoc 2017;6:e006044. 29021274 10.1161/JAHA.117.006044 PMC5721834

[2] Shroff GR, Bangalore S, Bhave NM, Chang TI, Garcia S, Mathew RO, et al Evaluation and management of aortic stenosis in chronic kidney disease: a scientific statement from the American Heart Association. Circulation 2021;143:e1088–e1114. 33980041 10.1161/CIR.0000000000000979

[3] Vahanian A, Beyersdorf F, Praz F, Milojevic M, Baldus S, Bauersachs J, et al 2021 ESC/EACTS guidelines for the management of valvular heart disease. Eur Heart J 2022;43:561–632. 34453165 10.1093/eurheartj/ehab395

[4] Maffeo D, Bettari L, Latib A, Maiandi C, Villa E, Messina A, et al Transfemoral transcatheter aortic valve replacement without contrast medium using the Medtronic CoreValve system: a single center experience. J Cardiovasc Surg (Torino) 2020;61:489–495. 10.23736/S0021-9509.20.11083-8 32241088

[5] Diaz Nuila ME, Gupta A, Alkhalil M. Single-access, non-contrast transcatheter aortic valve implantation, the ultimate minimalist approach: a case report. Eur Heart J Case Rep 2024;8:ytae040. 38332920 10.1093/ehjcr/ytae040 PMC10852022

[6] Mir T, Darmoch F, Ullah W, Sattar Y, Hakim Z, Pacha HM, et al Transcatheter versus surgical aortic valve replacement in renal transplant patients: a meta-analysis. Cardiol Res 2020;11:280–285. 32849962 10.14740/cr1092 PMC7430886

[7] Elhmidi Y, Bleiziffer S, Deutsch MA, Krane M, Mazzitelli D, Lange R, et al Acute kidney injury after transcatheter aortic valve implantation: incidence, predictors and impact on mortality. Arch Cardiovasc Dis 2014;107:133–139. 24556191 10.1016/j.acvd.2014.01.002

[8] Itelman E, Awesat J, Codner P, Aviv Y, Grinberg T, Abitbol M, et al Effects of contrast media on renal function following computed tomography prior to transcatheter aortic valve implantation. J Clin Med 2025;14:8754. 41464656 10.3390/jcm14248754 PMC12733380

[9] Jochheim D, Schneider VS, Schwarz F, Kupatt C, Lange P, Reiser M, et al Contrast-induced acute kidney injury after computed tomography prior to transcatheter aortic valve implantation. Clin Radiol 2014;69:1034–1038. 25017451 10.1016/j.crad.2014.05.106

[10] Herrmann HC, Mehran R, Blackman DJ, Bailey S, Mollmann H, Abdel-Wahab M, et al Self-expanding or balloon-expandable TAVR in patients with a small aortic annulus. N Engl J Med 2024;390:1959–1971. 38587261 10.1056/NEJMoa2312573

[11] Omari M, Durrani T, Diaz Nuila ME, Thompson A, Irvine T, Edwards R, et al Cardiac output in patients with small annuli undergoing transcatheter aortic valve implantation with self-expanding versus balloon expandable valve (COPS-TAVI). Cardiovasc Revasc Med 2024;73:15–22. 38955627 10.1016/j.carrev.2024.06.017

[12] Abdalwahab A, Omari M, Alkhalil M. Aortic valve intervention in patients with aortic stenosis and small annulus. Rev Cardiovasc Med 2025;26:26738. 40160595 10.31083/RCM26738 PMC11951497

[13] Okuno T, Asami M, Heg D, Lanz J, Praz F, Hagemeyer D, et al Impact of left ventricular outflow tract calcification on procedural outcomes after transcatheter aortic valve replacement. JACC Cardiovasc Interv 2020;13:1789–1799. 32763071 10.1016/j.jcin.2020.04.015

